# Accurate Quantification of AAV Vector Genomes by Quantitative PCR

**DOI:** 10.3390/genes12040601

**Published:** 2021-04-19

**Authors:** Cristina Martinez-Fernandez de la Camara, Michelle E. McClements, Robert E. MacLaren

**Affiliations:** 1 Nuffield Laboratory of Ophthalmology, Department of Clinical Neurosciences, John Radcliffe Hospital, Level 5&6, West Wing, Headley Way, Oxford OX3 9DU, UK; michelle.mcclements@eye.ox.ac.uk (M.E.M.); robert.maclaren@eye.ox.ac.uk (R.E.M.); 2 Oxford Eye Hospital, Oxford University Hospitals NHS Trust, John Radcliffe Hospital, West Wing, Headley Way, Oxford OX3 9DU, UK

**Keywords:** adeno-associated virus, gene therapy, quantitative PCR

## Abstract

The ability to accurately determine the dose of an adeno-associated viral (AAV) therapeutic vector is critical to the gene therapy process. Quantitative PCR (qPCR) is one of the common methods to quantify the AAV vector titre, but different variables can lead to inconsistent results. The aim of this study was to analyze the influence of the conformation of the DNA used as the standard control, and the enzymatic digestion was performed to release the viral genome from the protein capsid on the physical genome titration of a clinically relevant AAV8.RPGR vector, made to good laboratory practice standards in an academic setting. The results of this study showed that the conformation of the DNA used as standard has a significant impact on the accuracy of absolute quantification by qPCR. The use of supercoiled undigested plasmid DNA template generated a higher apparent titer, as compared to the use of linearized plasmid as the standard. In contrast to previous studies, the pre-treatment of the samples with Proteinase K, in addition to the high temperature step used after DNase I digestion, resulted in a reduction on AAV titers. Ideally, all AAV documentation should state which form of reference plasmid and which pre-treatment of the samples have been used to calculate titers, so that appropriate comparisons relating to dose toxicity and transduction efficacy can be made in the clinical scenario.

## 1. Introduction

Adeno-associated virus (AAV) is a small non-enveloped virus that depends on other viruses, such as adenovirus, herpes virus, or papilloma virus to complete its life cycle and replication. The AAV genome is a single-stranded DNA of approximately 4.7 kb that comprises two alternatively spliced viral genes enclosed within two symmetric inverted terminal repeats (ITRs): *rep*, required for the four viral replication proteins, and *cap*, which encodes the three AAV structural capsid proteins [1]. AAV have been widely used in gene therapy as vectors to transfer genes to humans due to its several advantages, such as its non-pathogenic nature, its ability to infect dividing and non-dividing cells, and the low probability to induce an inflammatory response. Recombinant AAV vectors are made by replacing the viral *rep* and *cap* genes with the gene of interest to be expressed in the target cells, hence the packaging capacity is limited and the relatively small size of the internal payload is limited to treat some diseases where the affected gene is too large to fit into the AAV vector [2,3,4].

Recombinant AAV vectors are arguably the best-characterized viral vectors in ophthalmology, with an exceptional safety profile being observed in several preclinical and clinical studies over more than 20 years [5,6,7,8,9,10]. The retina is an excellent target for gene therapy, since the accessibility allows for relatively non-invasive procedures, the effect of the treatment can be easily monitored, and the blood-retinal barrier limits the immunological response to the treatment by reducing systemic spread [11]. Recessive single gene retinal degenerations are the most amenable to be treated by gene therapy, because single gene supplementation can generally restore functional protein and ameliorate disease progression. Thus, the ideal delivery of the healthy copy of the gene would revert the phenotype without toxicity issues due to its overexpression. X-linked retinitis pigmentosa (XLRP) that is caused by mutations in *RPGR* is an ideal disease to be treated with AAV gene therapy because the size of the coding sequence fits well into AAV vectors (3.5 kb for RPGR^ORF15^). Additionally, the relatively high prevalence and severity of XLRP implies a favorable risk/benefit ratio for a gene therapy clinical trial [12]. Currently, there are three clinical trials (NCT03116113, NCT03252847, and NCT03316560) that evaluate the safety and efficacy of AAV vectors containing the human *RPGR* in subjects with XLRP that is caused by mutations in *RPGR* [9].

A requirement for the marketing of a gene therapy product is the characterization of the vector that must meet certain specifications that are related to vector identity, safety, purity, potency, and stability [13]. As part of the dosing and potency analysis of AAV vectors, the determination of the viral genome titer needs to be accurately quantified [14]. Quantitative real-time PCR (qPCR) is one of the analytical methods widely used in the quantification of AAV vectors because of its robustness and simplicity [15,16,17,18]. This method is a highly sensitive technique that allows for the amplification and the detection of DNA sequences in the same reaction. The detection is based on a fluorescence signal that is emitted by DNA-binding dyes or fluorescently labelled target-specific primers or probes. Quantitative data is derived for a determination of the cycle at which the amplification product signal crosses the detection threshold. The cycle number is proportional to the amount of sample material [19]. However, several factors can significantly impair the amplification efficiency of the DNA, such as secondary structures, preparation of the sample, or the topology of the DNA used as standard.

In this study, we analyzed the influence of the conformation of the DNA used for the standard curve and the enzymatic digestion that was performed during the sample preparation on the physical genome titration of a research grade AAV8.RPGR vector.

## 2. Materials and Methods

### 2.1. Preparation of Viral Vector

**Figure 1:**
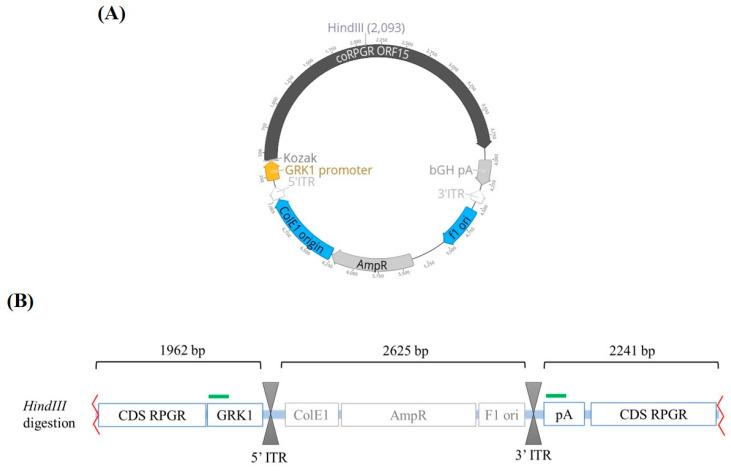
AAV.coRPGR plasmid used in this study. (**A**) Circular map for the AAV.coRPGR expression plasmid used to quantify the AAV8.RPGR titer by Quantitative real-time PCR (qPCR). This plasmid was previously used in a pre-clinical study [20] and contains the rhodopsin kinase promoter (GRK1) that will drive the expression of a codon optimized version of RPGR^ORF15^ followed by a polyadenylation signal. This transgene cassette is flanked by inverted terminal repeats (ITRs) from AAV2 required for replication and packaging. The restriction enzyme *HindIII* was used to linearize the plasmid. (**B**) Diagram showing the linearized plasmid following digestion with *HindIII* restriction enzyme. The green bars indicate the target sequences where the primers and probe bind, i.e., the GRK1 promoter and the bGH polyA (pA) sequence. Abbreviations: CDS; coding sequence, ITR; inverted terminal repeat, AmpR; ampicillin resistance gene.

Recombinant AAV2/8 vectors were produced by transient polyethylenimine (Sigma–Aldrich, Dorset, UK) co-transfection of HEK293 cells that were seeded in HYPERflasks (Corning, Flintshire, UK) using two plasmids: the AAV2 expression plasmid containing a codon optimised version of *RPGR^ORF15^* under the control of the rhodopsin kinase (GRK1) promoter (Figure 1A), and the commercial helper and packaging plasmid pDP8 (Plasmid Factory, Bielefeld, Germany) encoding Rep gene of AAV2 and Cap genes of AAV8. Viral vectors were purified using iodixanol gradient ultracentrifugation and they were concentrated by buffer exchange in Amicon 100K filters (Millipore, Dorset, UK).

### 2.2. AAV Sample Processing 

The first step in isolating the viral DNA involved treating each AAV sample with DNase I (New England Biolabs, Hitchin, UK) at 37 °C for 30 min to degrade any non-encapsidated DNA that may be present. To inactivate the DNase activity whilst also degrading the viral capsids, AAV samples were incubated at 95 °C for 10 min. Replicated samples were subjected to Proteinase K (Qiagen, Manchester, UK) digestion following the DNase I step to test whether the efficiency in releasing the encapsidated DNA could be increased by additional enzymatic treatment. These samples were incubated with Proteinase K at 50 °C for 60 min, followed by an inactivation at 95 °C for 20 min.

### 2.3. Analytical Standards for qPCR

Two standard curves were prepared for each qPCR reaction. One of the standard curves was made using the unmodified template plasmid, referred to as the supercoiled conformation. The second standard curve was made using a linearized form of the expression plasmid, prepared by digestion with the restriction enzyme *HindIII* (New England Biolabs, Hitchin, UK), which makes one cut (Figure 1B), by incubation at 37 °C for 1 h. The digestion reaction was tested in a 1% agarose gel, and pre-stained with 10% of ethidium bromide. The linearized plasmid was excised from the gel and then purified using the QIAquick Gel Extraction Kit (Qiagen, Manchester, UK), following the manufacturer’s instructions. DNA yield and quality were tested using the Invitrogen Qubit Fluorometer and the Qubit dsDNA High Sensitivity Assay Kit (Thermo Scientific, Northumberland, UK). 

Working solutions were prepared of supercoiled and linearized plasmid to obtain a final concentration of 1 ng/µL. These working solutions formed the first sample of each standard curve, from which a six-point 10-fold serial dilution from 1 ng/µL to 0.01 pg/µL was prepared. The number of copies per reaction was calculated using the online resource that was developed by Andrew Staroscik in the URI Genomics & Sequencing Center (https://cels.uri.edu/gsc/cndna.html, Accessed date: 14 October 2020). 

### 2.4. Quantitative PCR Analysis

Three independent qPCR reactions were carried out on CFX Connect Real-Time PCR Detection System from Biorad (Watford, United Kingdom) using the bovine GH poly adenylation (bGH polyA) signal and the rhodopsin kinase (GRK1) promoter as the target sequences. The sequence detection primers and the custom Taqman probe that were used in this study were synthetized by Life Technologies (Table 1). All of the PCRs were performed in a 20 µL final volume with 2 µL of sample DNA (diluted 1:100 and 1:1000), 900 nM of each primer, and 300 nM probe, and using TaqMan™ Fast Universal PCR Master Mix (2X) (Life Technologies, Cramlington, UK). All of the reactions were performed in triplicate and analyzed by both plasmid standards (supercoiled and linearized plasmid with *HindIII* enzyme,) run on the same plate. Both standard curves contained six serial dilutions of supercoiled or linearized plasmid standard (2.62 × 10^3^ to 2.62 × 10^8^ copies of plasmid DNA). A “no template DNA” was included as negative control. qPCR was performed with 1 cycle of 2 min at 50 °C, followed by one cycle of denaturation of 10 min at 95 °C, continued by 40 cycles of 15 s denaturation at 95 °C and 60 s annealing at 60 °C. 

### 2.5. Statistical Analysis

GraphPad Prism software (version 7.0, GraphPad Inc., San Diego, CA, USA) was used to analyze the data. Statistical evaluation of differences between standard curves and between the titers that were calculated using supercoiled or linearized plasmid was performed by the Mann–Whitney U test. Statistical evaluation of the differences between enzymatic pretreatments was performed by the Wilcoxon matched-pairs signed rank test.

## 3. Results

### 3.1. Supercoiled DNA Generates Higher Ct Values Than Linearized DNA

Repeatability between the technical replicates was highly consistent across the different dilutions in all of the standard curves, with the replicate variability falling within the set limit of <0.5 cycles. The coefficients of determination (r2) were all greater than 0.99 for each experiment (Table 2). These results indicated that the preparation of sample dilutions and assay performance were consistent, and the results were robust. 

The assay efficiency, calculated from the slope of the best fit straight line through the standard curve data points (E = (10(−1/slope)−1) × 100%), ranged between 89.4 and 110.2% (Table 2). No statistically significant difference was observed in amplification efficiencies between the different standard curves using supercoiled or linearized plasmid DNA as reference, using the GRK1 sequence, or the bGH polyA signal as targets (*p* > 0.05, Mann–Whitney test, *n* = 3). 

**Figure 2:**
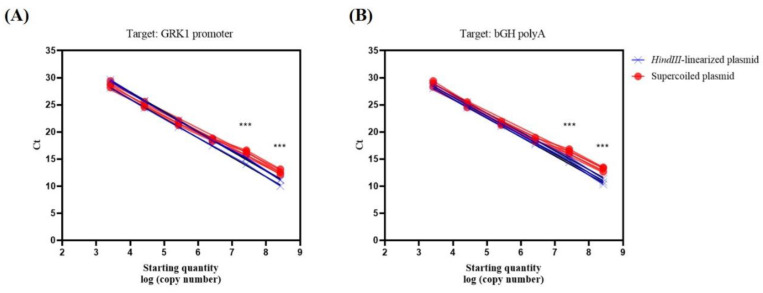
Standard curves obtained in three independent experiments targeting the GRK1 promoter (**A**) and the bGH polyA signal (**B**), using undigested plasmid (supercoiled) and linearized plasmid. Regardless of the target sequence, the Ct values between equimolar points of both standard curves are slightly different with higher values obtained using the supercoiled plasmid. These differences are statistically significant when higher starting quantities of DNA are used (*** *p* < 0.001, Mann–Whitney test, *n* = 3). The data shown in each graph represent the value for each technical replicate.

The threshold cycle numbers (Ct) for the supercoiled plasmid were higher as compared to the Ct values that were obtained with the linearized plasmid in both qPCR assays (GRK1 and bGH polyA) (Table 2). These differences were statistically significant when higher copy numbers were used as starting quantity, i.e., 2.62 × 10^7^ and 2.62 × 10^8^ (*p* < 0.001, Mann–Whitney test, *n* = 3) (Figure 2). 

### 3.2. Additional Treatment with Proteinase K Significantly Influences AAV Titre

**Figure 3:**
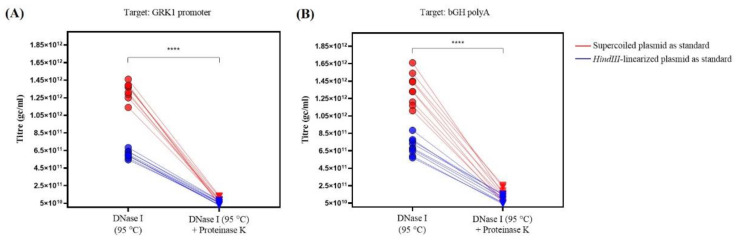
Graph representing the titer (gc/mL) calculated using the different DNA standards (supercoiled (red) vs. *HindIII*-linearized (blue)), on both qPCR assays (GRK1 promoter (**A**) and bGH polyA (**B**)) for the AAV sample treated with DNase I versus same sample with the additional Proteinase K treatment. For all of the conditions tested, the titer was significantly lower in samples treated with both enzymes (**** *p* < 0.0001, Wilcoxon matched-pairs signed rank test; W = −171.0, *n* = 18 (**A**,**B**)).

The AAV samples were processed in duplicate prior to PCR to evaluate the influence of the additional enzymatic treatment with Proteinase K to degrade the viral capsid on the quantification of the vector-derived DNA: thermal degradation at 95 °C after DNase I treatment with and without Proteinase K, as described in Methods. For both of the assays, the titer values were significantly lower when the samples were prepared with a combination of both treatments (DNase I and Proteinase K) (Figure 3). The additional digestion with Proteinase K also increases the variability—the coefficient of variation (CV) for all the assays performed with this pre-treatment ranged from 37.7% to 50.0%, whilst the CV for the data that were obtained with the single DNase I treatment ranged between 7.0 and 14.2% (Table 3). 

### 3.3. The Conformation of the Reference DNA Influences the AAV Titre

The following data investigate the difference in AAV titer depending on the structure of the template plasmid that was used as standard, i.e., undigested plasmid (supercoiled) or plasmid digested with *HindIII* (linearized). The amplification for all of the samples was within the range of the dilution series of the calibration curves. The average of Ct and standard quantification for the triplicates of each sample were calculated using both calibration curves (supercoiled versus linearized) that were included in each experiment. The AAV titer was calculated as the genome copies per milliliter (gc/mL) when considering the dilution factor of each sample and the volume of vector per reaction. 

The use of a *HindIII*-linearized plasmid as a standard resulted in lower AAV titers as compared to the use of supercoiled plasmid as standard (Table 3). The absolute difference in the AAV titer values for samples that were treated with DNase I is around 2, with significantly higher titers using supercoiled standard as reference as compared to the titers obtained using the linearized plasmid with *HindIII* as standard. The use of supercoiled plasmid as standard provided a significantly greater titer when compared to the use of *HindIII*-linearized plasmid (*p* < 0.0001, Mann–Whitney test, U = 0) for both target regions, with a ratio of 2.20 ± 0.03 and 1.94 ± 0.03 when targeting the GRK1 promoter and the bGH polyA, respectively. When the AAV sample was prepared with the additional Proteinase K treatment, the titers that were calculated with the supercoiled plasmid as standard were higher when compared to the values obtained using the *HindIII*-linearized plasmid as standard, although these differences are not statistically significant. Regardless of the plasmid form used as standard, there is no statistically significant difference in the AAV titer value when targeting the GRK1 promoter or the bGH polyA region (*p* > 0.05, Mann–Whitney test).

## 4. Discussion

AAV-based vectors have been widely used in gene therapy in animal models and human clinical trials due to their high efficiency driving a long-lasting transgene expression and its low immunogenicity and pathogenicity [21,22]. Despite their good safety profile, evidence indicates that AAV vectors can trigger an inflammatory response in different tissues, including immune privileged organs, such as the eye [23,24,25,26,27]. One of the main factors that can contribute to toxicity is the vector dose. The administration of high AAV vector doses can cause immunotoxicity that can be attributable to the viral capsid and/or to the transgene product [27,28]. The determination of the minimal effective and non-toxic dose of a candidate vector in animal models before clinical trials is of vital importance in ensuring the success of gene therapy. In addition to preventing toxicity issues, having an accurate and precise AAV titer is essential to define the actual dose that is administered to the animal models in pre-clinical studies and patients during all stages of clinical trials since this dose is often used as the input for other assays that are critical for determining product efficacy and/or safety, such as potency and infectious titer assays. Without an accurate assay, it is not possible to correctly control the dose that subjects are given and can make the data generated from potency assays variable and difficult to interpret. To that effect, the accurate and reliable titration of the recombinant AAV particles is an essential prerequisite. 

Quantitative PCR is a sensitive method for quantifying AAV titer available to most research facilities. However, the accuracy of this method will depend on the experimental design and performance. The first step is to extract the viral DNA from the AAV particles through the enzymatic digestion of the capsid or the use of high temperatures. The AAV8 capsid, which has a melting temperature of approximately 70 °C, can be efficiently degraded at 95 °C without the need to proceed with an enzymatic digestion [16,29,30]. In this study, we tested whether the combination of high temperature (95 °C) and the enzymatic digestion with proteinase K, was more efficient in releasing the DNA than a single heating step. Unlike other research that was carried out in this area, the results that were obtained in this study showed that the use of proteinase K to degrade the proteins of the viral capsid resulted in significantly lower titer values than the single treatment with DNase I, followed by an inactivation step at 95 °C. This was a surprising finding and contradictory to other studies that have shown an increase or an unchanged vector titer when using proteinase K treatment [16,31]. Nevertheless, the observed titer difference between supercoiled and linearized plasmid standards was maintained. It is possible that the proteinase K was not sufficiently inactivated in this study, as SDS is commonly applied to achieve this, whereas our protocol relied on heat inactivation. Identifying the specific cause of the difference would require extensive further investigations, but, for now, it is of interest to note that this appears to be the first presentation of such a finding. 

The conformation of the DNA used to elaborate the standard curves is one of the variables that can impact the qPCR assay performance. The standard curve included in a qPCR assay is calculated using two variables: the slope, which is a measure of the reaction efficiency, and the Y-intercept, which is the theoretical limit of detection of the reaction. Plasmid DNA is used as material for the calibration curves in qPCR. The supercoiled topological form that is characteristic of a plasmid DNA molecule is a twisted and winded circular DNA molecule that may exist in variable conformations, which would likely alter at different temperatures. This could influence the access of the PCR primers to the target sequence and, therefore, cause variation in the efficiency of primer binding and, consequently, target amplification. This could result not only in increased variability, but also in a lower amount of DNA that is accessible for each round of amplification, which has an impact on the standard curve parameters with a similar slope, but usually higher Ct values. 

Hence, the quantification of the viral vector-derived DNA using a calibration curve that was prepared with a DNA whose conformation is supercoiled or twisted in a way that makes the target regions less accessible would lead to less reliable results. Indeed, overestimation in qPCR has been reported that is caused by using supercoiled DNA as calibration standards [32,33]. The topology of this molecule prevents the correct performance of the qPCR, which, besides causing an overestimation in the absolute titers, contributes to increasing the variability in the results. This variability not only leads to the inaccuracy of AAV titer calculations, but also to the impossibility to cross-compare individual AAV samples using a correction factor.

In this study, we assessed the physical titer of an AAV.RPGR vector by qPCR using undigested plasmid (supercoiled conformation) and plasmid digested with the restriction enzyme *HindIII*. This enzyme makes one cut in the plasmid DNA, with the consequent linearization of the plasmid. Significant differences are observed when the use of supercoiled plasmid or the *HindIII*-linearized plasmid as standard are used. For both qPCR assays (GRK1 and bGH polyA), the titer values for the same AAV sample are significantly higher when using the supercoiled plasmid as standard. In accordance with other studies, the viral genome titers are overestimated when using supercoiled plasmid as the standard. The results obtained in this study suggest that the use of supercoiled plasmid as standard not only can affect the accuracy of the measure, but also the reproducibility of the results. It is critical to reliably and accurately titer AAV preparations to ensure appropriate dosing is achieved in clinical trials and the treatment of patients.

## 5. Conclusions

The structure of the DNA reference plasmid influences absolute quantification by qPCR of the control sample, thereby generating different AAV8.RPGR titres. One possible reason for this discrepancy is that linearized DNA may be easier for primers to bind to compared with supercoiled plasmids. Since the DNA in AAV is also linear after extraction, the linearized plasmid reference may provide a more accurate titre of AAV. All AAV publications should state which form of reference plasmid has been used to calculate titres, so that appropriate comparisons relating to dose toxicity and transduction efficacy can be made.

## Figures and Tables

**Table 1:** Primers and probes designed to quantify AAV.RPGR titre.

Target	Label	Sequence (5′→3′)
GRK1 promoter	Forward primer	TCTCTTAAGGTAGCCCCGG
	Reverse primer	ATCCGATTAGATCATTCTGCCC
	Taqman Probe (FAM)	CCTCACTTTTCCCCTGAGAAGGACA
bGH polyA	Forward primer	CTCGACTGTGCCTTCTAGTTG
	Reverse primer	ACCTACTCAGACAATGCGATG
	Taqman Probe (FAM)	TGCCACTCCCACTGTCCTTTCC

**Table 2:** PCR efficiency and performance on undigested and digested plasmid DNA.

	GRK1 Promoter		bGH polyA	
	Supercoiled	*HindIII*-Linearized	Supercoiled	*HindIII*-Linearized
Ct range	12.66–28.87	10.72–28.91	13.21–28.91	10.81–28.53
R square	0.9953	0.9993	0.9923	0.9989
Slope	−3.153	−3.607	−3.080	−3.487
Intercept	39.13	41.31	38.81	40.42
Efficiency	107.6%	89.4%	110.2%	93.5%

**Table 3:** Genome copies per milliliter (gc/mL) calculated in average for each sample, from three independent experiments.

Sample Treatment	Target	AAV Titre (gc/mL)		Significance (*p* Value)	Ratio (Supercoiled/Linearized)
		Supercoiled Plasmid as Standard	*HindIII*-Linearized Plasmid as Standard		
DNase I (95 °C)	GRK1 promoter	1.33 ± 0.03 × 10^12^ (7.1%)	6.06 ± 0.14 × 10^11^ (7.0%)	**** (*p* < 0.0001)	2.20 ± 0.03
	bGH polyA	1.36 ± 0.06 × 10^12^ (13.3%)	7.02 ±0.33 × 10^11^ (14.2%)	**** (*p* < 0.0001)	1.94 ± 0.03
DNase I (95 °C) +Proteinase K	GRK1 promoter	8.21 ± 1.11 × 10^10^ (40.6%)	5.42 ± 0.68 × 10^10^ (37.7%)	ns (*p* = 0.063)	1.50 ± 0.02
	bGH polyA	1.44 ± 0.24 × 10^11^ (50.0%)	9.51 ± 1.37 × 10^10^ (43.3%)	ns (*p* = 0.162)	1.48 ± 0.04

Values are the mean ± standard error of the mean. Values in parenthesis are the coefficients of variation. Statistically significant different titer values between calculations using supercoiled plasmid or *HindIII*-digested plasmid as standard are indicated (ns: not significant). The ratio indicates the absolute difference using supercoiled standard when compared to the *HindIII*-linearized plasmid (mean ± standard error of the mean).

## Data Availability

Data is contained within the article.
